# Sensory Properties, Textural Analysis, and Some Physical Analysis of Enriched Extruded Products Produced from Different Grain Products

**DOI:** 10.1007/s11130-025-01336-5

**Published:** 2025-03-18

**Authors:** Hayriye Genç, Nizam Mustafa Nizamlıoğlu

**Affiliations:** Department of Food Engineering, Faculty of Engineering, Karamanoglu Mehmetbey University, Karaman, 70200 Turkey

**Keywords:** Extruder, Whole wheat, Corn semolina, Textur, Expansion index

## Abstract

A functional product has been produced from a mixture of whole wheat flour and corn semolina, which can be consumed as a healthy snack compared to oily and additive-laden chip products. Extruded products were obtained at various output die temperatures (130, 150, and 170 °C) in a twin-screw extruder from mixtures prepared with different raw material humidity (12, 14, and 16%) from the mixtures of whole wheat flour, corn semolina, and D-glucose. The water binding capacity increased with increasing raw material humidity and output die temperature. Expansion index value was founded corn semolina had the highest value (3.11 ± 0.22), whereas whole wheat flour with D-glucose added had the lowest value (2.10 ± 0.34). Through sensory investigation, the most popular product groupings were determined to be whole wheat flour and corn semolina-extruded products. It is expected that this study will be able to produce extruded products from whole wheat flour and corn semolina and will set an example for the development of new functional products.

## Introduction

Extrusion cooking technology involves kneading, heating, and heat treatment applied at high temperatures for short periods [1]. Starch gelatinizes throughout the extrusion process, and its properties, such as elasticity, gas retention, dough characteristics, and gluten development [2, 3]. The biggest advantages of extrusion cooking technology are that it provides versatile working opportunities, has a very high production capacity, and increases the shelf life of products compared to other food processing methods [4–6].

To develop products that can vary in cell structure, shape, texture, and density, a wide range of raw materials from various sources and different compositions can be fed into the same extruder. Mechanical and thermal energy compresses/presses food powders to transform them into a viscoelastic liquid. Therefore, the characterization of the raw material is important for food extrusion. These are composition (moisture, protein, and fat content), particle size, surface friction, hardness, and stickiness [7]. Corn semolina, wheat semolina, etc., along with the main raw materials, the additives added in generally low amounts, play an important role in the extrusion and extruded product properties. These additives can serve as softeners, lubricants, binders, nutrient/supplementing agents, and expansion agents, or simply as flavor enhancers [8]. The extrusion of raw materials containing starch, protein, and lipids leads to many physical and chemical transformations that cause changes in the properties of the extruded products [9].

The amount of expansion is an important quality criterion for the textural, functional, and sensory properties of the extruded products [6]. The expansion index is the expansion phenomenon occurring due to pressure differences in the extruder process [10]. It has been reported in many studies that there is an inverse proportion between bulk density and expansion index [2, 11]. Water absorption capacity of extruded products increases up to certain temperature ranges and decreases as the temperature increases [12]. Water solubility is used to determine the number of soluble molecules as a result of starch dextrinization [13]. Texture analysis provides information about the texture and freshness of extruded products. The texture of extruded products is affected by screw speed, the properties of the components, humidity, and temperature [2]. Extruded products are required to have low hardness and breakability [3, 13].

The consumption of cereal-based dietary fibers and the use of extrusion technology, which provides ease in production while enriching with cereal-based fibers, have been highlighted, and the aim has been to investigate consumer preferences for the produced product. In this study, the effects of system parameters and raw material components on the physical, textural, and sensory properties of the final product in the extrusion cooking process were investigated. Extruded products were produced using corn semolina, whole wheat flour and D-glucose monohydrate at three different raw material humidity contents and three different output die temperatures.

## Materials and Methods

The research utilized corn semolina from ELİSA GOLD A.Ş. in Karaman, Turkey, wheat from POLAT Tarım Ürünleri in Konya, Turkey, and analytical grade D-glucose monohydrate from Merck in Germany.

### Preparation of Raw Material for Production

The wheats were ground in a laboratory-type roller flour mill to obtain whole wheat flour for the production (CHOPIN Technologies). Whole wheat flour, corn semolina, and D-glucose monohydrate were combined in a mixer (KMC510, Kenwood, UK), yielding six distinct formulations at varying ratios (Whole Wheat Flour: Corn Semolina; 100:0,70:30,0:100/and 5% glucose was added to each of the three mixtures separately). In order to obtain a homogeneous product from the formulations obtained, the moisture levels (12%, 14%, and 16%) were adjusted the day before by annealing (Table 1).

**Table 1:** Extruded product formulations and working conditions

Formulations	Whole Wheat Flour + Corn Semolina ratios (%w/w)	D-glucose monohydrate (w/w)
Whole Wheat Flour (WWF)	100%	-Not added
Whole Wheat Flour + Corn Semolina (WWF-CS)	70%+30%	- Not added
Corn Semolina Extruded Product (CS)	100%	-Not added
Whole Wheat Flour + D-glucose (WWF-D)	100%	+ 5% (added)
Whole Wheat Flour + Semolina Mixture + D-glucose (WWF-CS-D)	70%+30%	+ 5% (added)
Corn Semolina Extruded Product + D-glucose (CS-D)	100%	+ 5% (added)
**Extruder working conditions**		
Feed Raw Materials Moisture	12–14–16%	
Output Die Temperature	130–150 °C − 170 °C	
Samples Feed Speed	1,5 kg/h	
Screw Speed	250 rpm	
Exit Die Diameter	4 mm	
Screw Length/Diameter Ratio	40/1 (L/D)	
Barrel Diameter	21 mm	
Extruder 5 Section Temperature, Respectively	40–50 °C − 60–90 °C − 105 °C	

### Extruder Working Conditions and Production of Extrusion Products

By varying the extruder output die temperature (130, 150, and 170 °C), extruded products with various formulas and moisture content were produced. The extruded products were obtained using a laboratory-type, turn-same-direction, twin-screw, computer-controlled extruder (Rondol Technology, England). The resulting extruded products were dried in an airflow drying cabinet (Biyosan Kimya Laboratory Devices, Turkey) with a temperature setting of 50 °C to achieve a moisture content of 4–5%. The dried extruded products were placed in polyethylene bags and stored in a refrigerator until they were used for the chemical and physical analysis. The extruder working conditions are given in Table 1.

### Determination of Moisture

The moisture content of whole wheat flour, corn semolina, and extruded products was determined by drying in an oven (NUVE, FN055) at 135 °C for 2 h [14]. The moisture values of the extruded products were measured immediately after leaving the barrel of the extruder and cooling.

### Expansion Index

The diameter of each extruded product was measured three times using a caliper at three different points for each formulation. The expansion index values were calculated by dividing the obtained results by the diameter of the extruder’s exit die (4 mm) [3].

### Bulk Density, Real Density (ρt) and Porosity

A one-liter measuring cylinder was filled just above the liter mark and tapped twelve times to place the products up to the liter mark, and then the weights of the products were measured. Bulk density was calculated by the ratio of the obtained weight to volume. Real density was determined using a helium gas pycnometer (Micromeritics Accupyc II 1340 Gas Pycnometer, USA) and given as g/cm3 [15]. Using the actual density and bulk density values measured by the gas pycnometer, the porosity was calculated according to the formula below [16].

1 $$Po{\rm{ }} = {\rm{ }}1{\rm{ }}-{\rm{ }}\left({{\rm{\rho b / \rho t}}} \right)$$

ρb: Bulk Density (g/cm3), ρt: Real Density (g/cm3)

### Water Solubility and Water Binding Capacity

The ground extruded products (355 μm sieve) were weighed one gram (M1) into falcon tubes, 15 mL of distilled water was added, and centrifuged at 4000 rpm for 10 min after vortexing 8 times for 15 s at 5-minute intervals. The supernatant was transferred to the drying vessel (M2) and dried in an oven at 100 °C for one night. The part remaining in the tube was transferred to the drying container (M3) and kept in an oven at 100 °C for one night (M4). Solubility and water binding capacity were calculated according to the formula below [17].

2 $${\rm{Resolution }}\left({\rm{\% }} \right){\rm{ = }}\left({{{{\rm{M2 - Drying\,Container\,Tare}}} \over {{\rm{M1}}}}} \right){\rm{ \times 100}}$$

3 $$\eqalign{& {\rm{Water\,Binding }}\left({\rm{\% }} \right){\rm{ = }} \cr& {\rm{ }}\left({{{{\rm{M3 - }}\left({{\rm{M4 - Drying\,Container\,Tare}}} \right)} \over {{\rm{M1}}}}} \right){\rm{ \times 100}} \cr} $$

### Texture Analysis

Analysis was performed using the TA-XT2i Texture Analyzer (Texture Technologies Corp., Scarsdale, NY/Stable Micro Systems, Godalming, Survey, UK), a five-blade cracker cutter (HDP/KS5, Code: 12915). A 50 kg load cell was attached to the device, and height and force calibrations were made. The pre-test, test, and post-test speeds were 2.0, 2.0, and 2.0 mm/s, respectively. The tension (60%) target mode and trigger force were 5.0 g. In the analysis of extruded products, a Kramer 5-blade cutting probe was used. All samples analyzed were 7 mm in length, and 10 repeated analyses were conducted [4]. Brittleness is determined by the average number of peaks on the force deformation curve in extruded products [8]. Fragility is defined as the distance (mm) required to break extruded products [4, 5].

### Sensory Analysis

Sensory analyses of extruded products were carried out by a educated panel group of 10 people aged between 20 and 50, consisting of Karamanoğlu Mehmetbey University Faculty of Engineering Food Engineering students and lecturers [18]. Randomly served chip samples were subjected to sensory evaluation for color/appearance, taste/smell, hardness/chewyness, crispness, brittleness, pore structure, and overall acceptability. The panelists were asked to evaluate the samples on a 5-scale hedonic-type scale. On the hedonic scale, 5 indicates the highest score, while 1 indicates the lowest.

### Statistical Analysis

All study results were analyzed using MINITAB release 19.0’s factorial trial design variance analysis technique, and differences between groups were determined using Tukey’s multiple comparison test. All results were expressed as the mean and standard deviation of the five independent determinations. The results were expressed as mean ± Stdev (standard deviation), and the significance level was set at *P* < 0.05.

## Results and Discussion

### Moisture, Water Solubility and Water Absorption Results

The moisture content of the fed mixture is the most critical factor influencing the volume and structure of the extruded product [19]. The highest moisture content was found in extruded products produced at CS-D 16% raw material humidity and 130 °C output die temperature, while the lowest moisture content was found in WWF-CS 12% raw material humidity and 170 °C output die temperature (Table 2). Extruded products produced with WWF had a high moisture content (9.45 ± 1.54%), while CS and WWF-CS extruded products had a low moisture content (7.48 ± 1.71 and 7.66 ± 0.69%, respectively). Extruded products containing D-glucose monohydrate (WWF-D, M-D, and WWF-CS-D) were determined to have a higher moisture value compared to WWF, CS, and WWF-CS extruded products. The moisture content of extruded products has been found to increase with the addition of 5% D-glucose monohydrate (*p* < 0.05). The change between the extruder output die temperature and the moisture content of the extruded products was found to be statistically insignificant. Researchers determined that the difference in moisture values in extruded products was due to different raw material humidity (corn semolina and bulgur flour; 10–14%) [6] and the difference in extrusion processing conditions (raw material humidity, screw speed, and feed rate) [10].

**Table 2:** Moisture, water solubility and water absorption results of extruded products produced with different combinations of whole wheat flour, corn semolina and D-glucose at different raw materials humiditys and output die temperatures

Products	Moisture (%)	Temperature (°C)	Water absorption (%)	Solubility in water (%)	Water binding capacity (%)	Moisture (%)
WWF	12	130	3,38 ± 0,042st	27,20 ± 0,171f-n	354,24 ± 1,937r-u	7,81 ± 0,109tu
WWF	12	150	3,42 ± 0,013rst	25,60 ± 0,284 h-p	354,49 ± 3,316r-u	7,69 ± 0,196u
WWF	12	170	3,29 ± 0,017t	26,39 ± 0,131 g-o	342,08 ± 4,605u	7,84 ± 0,143stu
WWF	14	130	3,62 ± 0,024qrs	25,25 ± 0,416ı-p	374,20 ± 4,533n-u	8,91 ± 0,074o-u
WWF	14	150	3,69 ± 0,037pqr	22,38 ± 0322n-r	408,38 ± 1,512 g-n	9,09 ± 0,459o-u
WWF	14	170	3,70 ± 0,036pq	18,76 ± 0,148qr	469,32 ± 9,130 cd	9,61 ± 0,121 L-r
WWF	16	130	3,95 ± 0,083 m-p	21,80 ± 0,727o-r	426,41 ± 0,113e-j	11,27 ± 0,239 g-l
WWF	16	150	3,96 ± 0,032 m-p	18,54 ± 0,324r	453,33 ± 6,642c-f	11,30 ± 0,533 g-l
WWF	16	170	3,83 ± 0,031n-q	18,53 ± 0,241r	355,50 ± 7,326r-u	11,61 ± 0,054f-ı
CS	12	130	5,37 ± 0,062b-e	31,09 ± 1,077b-g	438,79 ± 9,832d-h	5,26 ± 0,206v
CS	12	150	5,48 ± 0,070abc	31,96 ± 0,654a-f	435,58 ± 3,428d-ı	5,37 ± 0,429v
CS	12	170	5,31 ± 0,080b-e	28,26 ± 0,354c-l	456,89 ± 9,404cde	5,38 ± 1,045v
CS	14	130	5,26 ± 0,065cde	28,38 ± 1,670c-l	482,70 ± 6,191bc	8,03 ± 0,175q-u
CS	14	150	5,45 ± 0,049a-d	23,67 ± 2,583 L-q	519,73 ± 9,030ab	7,95 ± 0,052r-u
CS	14	170	5,69 ± 0,052a	24,39 ± 0,137j-p	543,09 ± 5,228a	7,69 ± 0,217u
CS	16	130	5,38 ± 0,073b-e	32,25 ± 1,051a-d	440,41 ± 7,851d-g	8,60 ± 0,491p-u
CS	16	150	5,18 ± 0,078def	23,07 ± 0,694 m-r	528,27 ± 2,975a	9,48 ± 0,621n-t
CS	16	170	5,70 ± 0,062a	24,12 ± 0,845k-p	527,16 ± 2,354a	9,57 ± 0,430 m-r
WWF-CS	12	130	3,73 ± 0,011opq	29,99 ± 1,427b-ı	356,63 ± 5,959r-u	4,39 ± 0,121v
WWF-CS	12	150	3,89 ± 0,182 m-q	29,45 ± 0,738b-ı	367,53 ± 7,657o-u	5,65 ± 0,208v
WWF-CS	12	170	3,75 ± 0,030opq	28,64 ± 0,966c-k	383,67 ± 1,001 L-s	3,99 ± 0,147v
WWF-CS	14	130	4,29 ± 0,066jk	27,44 ± 0,783d-m	406,43 ± 2,898 g-o	8,40 ± 0,309p-u
WWF-CS	14	150	4,30 ± 0,082jk	27,46 ± 0,872d-m	409,03 ± 2,878 g-n	8,60 ± 0,096p-u
WWF-CS	14	170	4,29 ± 0,050jk	32,27 ± 0,058a-d	385,24 ± 2,098k-s	7,92 ± 0,092r-u
WWF-CS	16	130	4,38 ± 0,051ıj	27,32 ± 2,616e-m	397,68 ± 5,285ı-q	9,98 ± 0,860ı-p
WWF-CS	16	150	4,66 ± 0,028hı	27,93 ± 2,974c-m	399,80 ± 3,470 h-q	10,53 ± 0,617 h-o
WWF-CS	16	170	4,63 ± 0,021hı	27,49 ± 1,436d-m	400,90 ± 6,164 g-p	9,52 ± 0,267n-s
WWF-D	12	130	3,88 ± 0,174 m-q	31,74 ± 0,886a-f	343,41 ± 5,453tu	8,45 ± 0,308p-u
WWF-D	12	150	4,67 ± 0,056 h	26,37 ± 0,286 g-o	356,96 ± 2,401r-u	9,08 ± 0,332o-u
WWF-D	12	170	3,77 ± 0,078opq	24,14 ± 0,181k-p	355,44 ± 1,960r-u	9,71 ± 0,826k-q
WWF-D	14	130	3,86 ± 0,071n-q	25,49 ± 0,292 h-p	373,90 ± 5,649n-u	11,89 ± 0,066e-h
WWF-D	14	150	3,83 ± 0,013n-q	23,28 ± 0,274 m-r	364,70 ± 1,811p-u	11,55 ± 0,359f-j
WWF-D	14	170	3,64 ± 0,070qrs	21,66 ± 0,175o-r	357,89 ± 1,632r-u	11,07 ± 0,612 g-n
WWF-D	16	130	3,87 ± 0,039 m-q	24,03 ± 0,351k-p	381,67 ± 3,912 L-t	12,17 ± 0,308d-h
WWF-D	16	150	3,87 ± 0,085 m-q	20,96 ± 0,808pqr	383,05 ± 8,207 L-s	13,69 ± 0,193a-d
WWF-D	16	170	3,99 ± 0,088 L-o	20,91 ± 0,289pqr	368,15 ± 2,999o-u	13,75 ± 0,208a-d
CS-D	12	130	4,61 ± 0,006hı	33,79 ± 4,309ab	401,82 ± 8,942 g-p	10,55 ± 0,052 h-o
CS-D	12	150	5,19 ± 0,042def	36,30 ± 0,523a	388,14 ± 9,392j-r	11,25 ± 0,387 g-m
CS-D	12	170	4,97 ± 0,048 fg	32,50 ± 0,967abc	387,60 ± 3,507j-r	10,52 ± 0,395 h-o
CS-D	14	130	5,12 ± 0,093efg	29,20 ± 4,293b-j	418,15 ± 9,935e-l	13,32 ± 0,003a-e
CS-D	14	150	5,37 ± 0,096b-e	31,94 ± 1,160a-f	423,54 ± 9,156e-k	12,60 ± 0,296b-g
CS-D	14	170	5,59 ± 0,085ab	32,21 ± 0,428a-e	434,58 ± 8,250d-ı	13,07 ± 0,443a-f
CS-D	16	130	4,85 ± 0,078gh	31,46 ± 2,864a-f	377,20 ± 9,915 m-u	14,69 ± 0,076a
CS-D	16	150	4,88 ± 0,150gh	30,98 ± 0,250b-g	438,45 ± 9,330d-h	12,29 ± 0,760c-g
CS-D	16	170	4,68 ± 0,220 h	32,13 ± 0,615a-e	415,35 ± 8,169f-m	11,88 ± 1,874e-h
WWF-CS-D	12	130	3,93 ± 0,068 m-p	28,83 ± 1,405c-k	373,35 ± 9,714n-u	9,70 ± 0,154k-q
WWF-CS-D	12	150	3,91 ± 0,032 m-q	30,18 ± 0,616b-ı	374,60 ± 4,873n-u	9,99 ± 0,632ı-p
WWF-CS-D	12	170	3,83 ± 0,022n-q	28,88 ± 2,228b-k	371,34 ± 5,833n-u	9,87 ± 0,675j-p
WWF-CS-D	14	130	4,31 ± 0,252jk	27,93 ± 1,783c-m	361,11 ± 3,716q-u	11,38 ± 0,219 g-k
WWF-CS-D	14	150	4,15 ± 0,079j-m	30,27 ± 0,827b-h	379,59 ± 5,330 L-u	11,13 ± 1,174 g-n
WWF-CS-D	14	170	3,90 ± 0,029 m-q	30,33 ± 1,240b-h	376,24 ± 8,087 m-u	12,27 ± 0,702c-g
WWF-CS-D	16	130	4,05 ± 0,138k-n	28,29 ± 2,141c-l	349,50 ± 3,153r-u	14,23 ± 0,129ab
WWF-CS-D	16	150	3,74 ± 0,044opq	31,27 ± 2,732b-g	351,54 ± 9,098r-u	13,89 ± 0,166abc
WWF-CS-D	16	170	4,25 ± 0,068jkl	29,76 ± 1,237b-ı	347,26 ± 2,019stu	13,61 ± 0,118a-d

*WWF: whole wheat flour extruded product, CS: corn semolina extruded product, D (%5, w/w): D-glucose monohydrate, WWF-CS: (70:30): 70% whole wheat flour and 30% corn semolina mixture

** Different letters show the means in the same column are significantly (*p* < 0.05)

Water solubility is the determination of the soluble molecular components released from starch [20]. The highest water solubility value, was found in the CS-D product produced at 12% raw material humidity and 150 °C die exit temperature, and the lowest water solubility value, was found in the extruded product produced at 16% raw material humidity and 170 °C die exit temperature (Table 2). Extruded product groups comprising corn semolina have been determined to be more water-soluble than extruded products containing whole wheat flour. WWF extruded products had the lowest water solubility (22.71 ± 3.41), whereas CS-D had the greatest (32.27 ± 2.69). The change in water solubility values of extruded products was found to be statistically significant (*p* < 0.05). Increasing raw material moisture and output die temperature has been detected to decrease the water solubility of extruded products. WWF-extruded products are supposed to have limited water solubility due to the high amount of bran used. Reserches reported that the water solubility of the extruded products produced from corn semolina [20], from mixtures of fenugreek, oat, and pea [21] and mixtures of corn grits and pea hulls [22] decreased with an increase in raw material humidity (18–20%). Hashimoto and Grassmann [23] and Yağcı and Göğüş [2] stated that the increase in the amount of bran and the rise in the output die temperature in the output die temperature (180–210 °C) reduced the water solubility in the extruded products.

Researchers have reported that temperature and humidity increases affect starch gelatinization and are the determining factors in water binding capacity [3, 11]. Chang and Ng [24], who obtained extruded products from wheat and ginseng blended formulations, reported that increasing raw material humidity (25–35%) and output die temperature (110–140 °C) increased the water binding capacity. In our research, both analyses were performed, and the results confirmed each other. The highest water binding capacity, was found in the CS extruded product produced at 14% raw material humidity and 170 °C output die temperature, and the lowest in the WWF extruded product, produced at 12% raw material humidity and 170 °C output die temperature (Table 2). Extruded products containing whole wheat flour and D-glucose monohydrate have been found to have a reducing effect on water binding capacity. Extruded product water binding capacity values varied significantly with formulation and raw material humidity (*p* < 0.05). Similar results are seen in the water absorption values in Table 2. Whole wheat flour and extruded products containing D-glucose are hypothesized to diminish starch’s water-binding capacity by inhibiting gelatinization. Sue et al. [25] produced extruded products using corn flour, rice flour, corn starch with potato starch (%30 w/w), and rice flour with potato starch (%30 w/w) with a screw speed of 75 rpm, a feed moisture of 25% (w/w), a barrel temperature ranging from 80 °C to 140 °C, and a die size of 1.88 mm. They reported that the water-binding capacities of extruded products increased with the rise in temperature but decreased in other extruded products except for corn flour at 140 °C. Sing et al. [26] extruded potato-rice and chickpea mixtures at different moisture contents (12.6–19.4%), screw speeds (349–601 rpm), and barrel temperatures (116–184 °C). They reported that the water binding capacity values of extruded products ranged between 4.06 and 5.0 g/g, that an increase in feed moisture led to a significant increase in water binding capacity, and that an increase in temperature caused a decrease.

### Expansion Index and Density Results

The expansion index plays an important role in the acceptability of extruded products. Extruded products made from maize flour are said to have a high expansion index due to their starch, amylose, and low fiber content [20, 27]. Extruded products produced at CS 12% raw material humidity and 130 °C output die temperature had the highest expansion index (3.38 mm), whereas extruded product created at WWF (16% raw material humidity and 170 °C output die temperature) had the lowest (1.21 mm). While a higher expansion index (3.11 mm) was observed in extruded products produced with CS, the expansion index was determined to be lower in WWF (2.13 mm) and WWF-D (2.10 mm) extruded products (Table 3). The addition of D-glucose monohydrate to CS and WWF-CS formulations lowered the extruded products’ expansion index values. As the moisture content increased, the expansion index decreased (*p* < 0.05). It can be seen that WWF-extruded products have the lowest expansion index. This is assumed to be due to the high fiber content of whole wheat flour. Masatcıoğlu [17] found that adding sugars (2% D-ribose and 2% D-glucose) to a mixture of white maize flour, soy protein isolate, and asparagine lowered the expansion index of the extruded product. The decrease in expansion in the final product with sugar content has been expressed as the combined effect of the reduction in pore growth and the increase in contraction occurring when the product leaves the die. Yuliani et al. [28] stated that the expansion index decreases as the output die temperature increases.

**Table 3:** Expansion index and density results of extruded products produced with different combinations of whole wheat flour, corn semolina and D-glucose at different raw materials humiditys and output die temperatures

Products	Moisture (%)	Temperature (°C)	Expansion index	Bulk density (g/cm3)	True density (g/cm3)	Porosity
WWF	12	130	2,28 ± 0,049 L-t	0,086 ± 0,0018abacad	1,266 ± 0,0015ad	0,932 ± 0,0014c-g
WWF	12	150	2,39 ± 0,034j-s	0,089 ± 0,0037aaabac	1,296 ± 0,0005ac	0,932 ± 0,0028c-g
WWF	12	170	2,34 ± 0,258k-s	0,099 ± 0,0043x-aa	1,342 ± 0,0027aa	0,926 ± 0,0033f-ı
WWF	14	130	2,19 ± 0,058o-v	0,100 ± 0,0017w-z	1,413 ± 0,0013st	0,929 ± 0,0013e-h
WWF	14	150	2,42 ± 0,052ı-s	0,116 ± 0,0026q-v	1,377 ± 0,0012y	0,916 ± 0,0018k-n
WWF	14	170	2,06 ± 0,094r-x	0,121 ± 0,0038p-t	1,413 ± 0,0030st	0,915 ± 0,0026k-n
WWF	16	130	2,21 ± 0,168o-v	0,145 ± 0,0022o	1,432 ± 0,00291	0,899 ± 0,0012op
WWF	16	150	2,09 ± 0,080q-w	0,156 ± 0,0035lmn	1,512 ± 0,0003a	0,897 ± 0,0023opq
WWF	16	170	1,22 ± 0,076y	0,272 ± 0,0031a	1,445 ± 0,0006p	0,812 ± 0,0021c
CS	12	130	3,38 ± 0,013a	0,075 ± 0,0002adae	1,199 ± 0,0022ah	0,938 ± 0,0003a-d
CS	12	150	3,28 ± 0,096ab	0,074 ± 0,0018adae	1,223 ± 0,0021af	0,939 ± 0,0014abc
CS	12	170	3,18 ± 0,152abc	0,071 ± 0,0005ae	1,105 ± 0,0025aı	0,935 ± 0,0004b-e
CS	14	130	2,97 ± 0,311b-f	0,107 ± 0,0008u-x	1,315 ± 0,0007ab	0,918 ± 0,0007ı-n
CS	14	150	3,16 ± 0,216abc	0,105 ± 0,0004v-y	1,399 ± 0,0018v	0,925 ± 0,0004 g-j
CS	14	170	3,18 ± 0,185abc	0,095 ± 0,0003y-ab	1,394 ± 0,0004vw	0,932 ± 0,0002c-g
CS	16	130	2,92 ± 0,224b-g	0,123 ± 0,0015pqr	1,433 ± 0,0008q	0,914 ± 0,0010k-n
CS	16	150	3,06 ± 0,251a-d	0,093 ± 0,0003zaaab	1,449 ± 0,0057op	0,936 ± 0,0002b-e
CS	16	170	2,91 ± 0,125b-g	0,120 ± 0,0035p-t	1,451 ± 0,0010no	0,917 ± 0,0025j-n
WWF-CS	12	130	3,04 ± 0,118a-e	0,077 ± 0,0012acadae	1,245 ± 0,0019ad	0,938 ± 0,0009a-d
WWF-CS	12	150	2,88 ± 0,115b-h	0,074 ± 0,0016ae	1,241 ± 0,0051e	0,941 ± 0,0012ab
WWF-CS	12	170	2,97 ± 0,085b-f	0,068 ± 0,0007ae	1,215 ± 0,0027ag	0,944 ± 0,0005a
WWF-CS	14	130	2,88 ± 0,103b-h	0,094 ± 0,0019y-ab	1,363 ± 0,0022z	0,931 ± 0,0014d-g
WWF-CS	14	150	2,72 ± 0,008d-k	0,089 ± 0,0016aaabac	1,342 ± 0,0036aa	0,934 ± 0,0013b-f
WWF-CS	14	170	2,64 ± 0,090e-l	0,094 ± 0,0008y-ab	1,310 ± 0,0034ab	0,928 ± 0,0005e-h
WWF-CS	16	130	2,50 ± 0,173 h-p	0,126 ± 0,0026pq	1,417 ± 0,0028st	0,911 ± 0,0017n
WWF-CS	16	150	2,48 ± 0,046 h-q	0,117 ± 0,0021q-u	1,419 ± 0,0016rs	0,918 ± 0,0015j-n
WWF-CS	16	170	2,33 ± 0,110k-s	0,122 ± 0,0017p-s	1,412 ± 0,0004t	0,913 ± 0,0012lmn
WWF-D	12	130	2,71 ± 0,074d-k	0,121 ± 0,0022p-t	1,413 ± 0,0006st	0,915 ± 0,0016k-n
WWF-D	12	150	2,57 ± 0,043 g-o	0,126 ± 0,0016pq	1,424 ± 0,0010r	0,911 ± 0,0011mn
WWF-D	12	170	2,21 ± 0,115n-v	0,146 ± 0,0018no	1,431 ± 0,0002q	0,898 ± 0,0013opq
WWF-D	14	130	2,22 ± 0,034 m-v	0,168 ± 0,0028kl	1,461 ± 0,0008jkl	0,885 ± 0,0019rs
WWF-D	14	150	2,02 ± 0,033s-x	0,180 ± 0,0022ıj	1,463 ± 0,0047ıjk	0,877 ± 0,0017st
WWF-D	14	170	1,83 ± 0,042vwx	0,201 ± 0,0017efg	1,462 ± 0,0003jkl	0,862 ± 0,0012vwx
WWF-D	16	130	1,89 ± 0,040t-x	0,211 ± 0,0009e	1,473 ± 0,0001efg	0,857 ± 0,0006x
WWF-D	16	150	1,78 ± 0,005wx	0,229 ± 0,0050d	1,475 ± 0,0005 deg	0,845 ± 0,0034yz
WWF-D	16	170	1,68 ± 0,065y	0,246 ± 0,0043c	1,471 ± 0,0009fgh	0,833 ± 0,0029aaab
CS-D	12	130	2,96 ± 0,095b-g	0,160 ± 0,0023 lm	1,466 ± 0,0010hıj	0,891 ± 0,0017pqr
CS-D	12	150	2,87 ± 0,149c-h	0,145 ± 0,0101o	1,459 ± 0,0009kl	0,901 ± 0,0070o
CS-D	12	170	2,61 ± 0,120f-n	0,129 ± 0,0008p	1,469 ± 0,0001ghı	0,912 ± 0,0006lmn
CS-D	14	130	2,46 ± 0,087ı-q	0,176 ± 0,0011jk	1,480 ± 0,0010bcd	0,881 ± 0,0008s
CS-D	14	150	2,33 ± 0,139k-s	0,189 ± 0,0105hı	1,471 ± 0,0005fgh	0,872 ± 0,0072tu
CS-D	14	170	2,43 ± 0,079ı-r	0,225 ± 0,0002d	1,477 ± 0,0006cde	0,848 ± 0,0001y
CS-D	16	130	2,23 ± 0,121 m-t	0,258 ± 0,0025b	1,483 ± 0,0003bc	0,826 ± 0,0016ab
CS-D	16	150	2,76 ± 0,017d-j	0,242 ± 0,0019c	1,486 ± 0,0003b	0,837 ± 0,0013zaa
CS-D	16	170	2,14 ± 0,184p-w	0,210 ± 0,0052e	1,476 ± 0,0003def	0,858 ± 0,0034x
WWF-CS-D	12	130	2,80 ± 0,052c-ı	0,109 ± 0,0049t-x	1,391 ± 0,0004wx	0,922 ± 0,0035 h-k
WWF-CS-D	12	150	2,62 ± 0,017f-m	0,111 ± 0,0031s-w	1,386 ± 0,0005x	0,920 ± 0,0022ı-l
WWF-CS-D	12	170	2,44 ± 0,111ı-r	0,113 ± 0,0041r-v	1,405 ± 0,0028u	0,919 ± 0,0031ı-m
WWF-CS-D	14	130	2,38 ± 0,068j-s	0,158 ± 0,0023 lm	1,451 ± 0,0004mno	0,891 ± 0,0016pqr
WWF-CS-D	14	150	2,18 ± 0,073o-w	0,152 ± 0,0018mno	1,456 ± 0,0008lmn	0,896 ± 0,0011opq
WWF-CS-D	14	170	2,12 ± 0,054p-w	0,159 ± 0,0028 lm	1,457 ± 0,0007 lm	0,891 ± 0,0019qr
WWF-CS-D	16	130	2,04 ± 0,047r-x	0,206 ± 0,0006ef	1,476 ± 0,0002def	0,861 ± 0,0004wx
WWF-CS-D	16	150	1,91 ± 0,010t-x	0,194 ± 0,0057fgh	1,471 ± 0,0002fgh	0,868 ± 0,0039uvw
WWF-CS-D	16	170	1,85 ± 0,044u-x	0,193 ± 0,0085gh	1,469 ± 0,0006ghı	0,869 ± 0,0058uv

*WWF: whole wheat flour extruded product, CS: corn semolina extruded product, D (%5, w/w): D-glucose monohydrate, WWF-CS: (70:30): 70% whole wheat flour and 30% corn semolina mixture

** Different letters show the means in the same column are significantly (*p* < 0.05)

Bulk density is one of the important parameters in extruded products and is associated with the expansion index [8]. Extruded products with a lower expansion index have a higher density, which is inversely proportional to the expansion index [26]. The WWF extruded product, produced at 16% raw material humidity and 170 C output die temperature, had the highest bulk density, while the WWF-CS extruded product, produced at 12% raw material humidity and 170 C output die temperature, had the lowest bulk density (*p* < 0.05) (Table 3). Bulk density was found to be high in CS-D extruded products but low in CS and WWF-CS extruded products (*p* < 0.05). The bulk density values were found to rise with the addition of D-glucose monohydrate. Korked et al. [29] stated that the bulk density increased with the increase in raw material humidity in the extruded products they produced from food by-products. Samray [3] stated that the bulk density of extruded products at 13% and 15% raw material humidity was lower than that of products at 17% raw material humidity.

Density expresses how much the unit volume of the product weighs and is a very important parameter in the production of extruded products [2, 8]. The WWF extruded product had the highest true density value at 16% raw material humidity and 150 °C output die temperature, while the CS extruded product had the lowest at 170 °C output die temperature and 12% raw material humidity (Table 3).

While CS-D (1.474 g/cm3) extruded products had a high general actual density value, CS (1.329 g/cm3) and WWF-CS (1.329 g/cm3) extruded products had a low actual density. The actual density values of extruded products containing D-glucose monohydrate were found to be greater than those of other extruded products. It has been determined that the actual density of products with a low expansion index is high. Sumargo et al. [30] and Ding et al. [11] stated that increase in the density of the extruded products with increasing raw material humidity.

A porous, inflated, and spongy structure can arise as a result of the quick release of pressure at the output die. The resulting porosity is a parameter that can be used to define the expansion feature of extruded products [31]. The WWF-CS extruded product produced at 12% raw material humidity and 170 °C output die temperature had the maximum porosity value, while the lowest was found in the WWF extruded product produced at 16% raw material humidity and 170 °C output die temperature. Among the extruded products produced with the different formulations, the highest porosity value was found in the CS (0.928) and WWF-CS (0.928) extruded products, and the lowest porosity value was found in the CS-D (0.869) extruded products. WWF, CS, and WWF-CS extruded products were found to be more porous than extruded products containing D-glucose monohydrate (*p* < 0.05). Porosity has been found to be directly proportional to the expansion index, and expanded products have high porosity. Pitts et al. [32] stated that the addition of sugar to extruded products a small pore size and a thickening of the cell wall. Yağcı and Göğüş [33] stated that increasing the temperature increases the porosity of extruded products. The researchers explained that the decrease in porosity at temperatures of 150 °C and above may be due to increased dextrinization and weakening of the structure [34].

### Texture Results

Brittleness is a widely used textural property for extruded products and is associated with the moisture content of the product [34]. The CS-D extruded product with the highest brittleness was produced at 12% raw material humidity and 150 °C output die temperature, while the WWF-D extruded product with the lowest brittleness was produced at 16% raw material humidity and 170 °C output die temperature. Among the different formulations, CS extruded products had the maximum brittleness (51.44), whereas WWF-D extruded products had the lowest brittleness (10.48). Extruded products containing wholemeal flour were found to have low crispness. The addition of maize semolina to whole wheat flour has been found to increase fragility (*p* < 0.05). Ding et al. [11] reported that the brittleness of the extruded products they produced from rice flour decreased with increasing raw material moisture (14–22%) and increased slightly with increasing die temperature (100–140 °C).

The product CS-D, produced at 14% raw material humidity and 150 °C output die temperature, was found to have the highest fragility, while the product CS extruded, produced at 12% raw material humidity and 150 °C output die temperature, was found to have the lowest fragility. After examining the fragility of extruded products made with various formulas, it was found that the products with the highest fragility were CS-D (1928.79), and the products with the lowest fragility were WWF-D (869.37). Products produced from corn semolina are found to have high brittleness and fragility values (Table 4). D-glucose monohydrate-containing extruded products have been found to be less fragile than other products (*p* < 0.05). Min et al. [35] stated that the brittleness of flaxseed and corn semolina-extruded products decreased significantly by increasing the raw material humidity and output die temperature.

**Table 4:** Texture results of extruded products produced with different combinations of whole wheat flour, corn semolina and D-glucose at different raw materials humiditys and output die temperatures

Products	Moisture (%)	Temperature (°C)	Hardness	Fragility	Brittleness
WWF	12	130	351,09 ± 8,65 lm	855,86 ± 22,42uv	22,67 ± 10,26 g-o
WWF	12	150	560,76 ± 9,23bc	1218,78 ± 13,11j	18,00 ± 1,73 h-p
WWF	12	170	579,60 ± 8,74b	1292,41 ± 11,24hı	25,00 ± 7,00f-l
WWF	14	130	614,63 ± 5,54a	1204,08 ± 28,62jk	12,33 ± 4,62k-p
WWF	14	150	551,74 ± 10,72 cd	1229,34 ± 26,72ıj	14,33 ± 2,52ı-p
WWF	14	170	522,16 ± 6,34efg	1104,85 ± 30,84mno	16,33 ± 3,06 h-p
WWF	16	130	617,77 ± 0,08a	774,50 ± 23,43x-aa	6,33 ± 3,21op
WWF	16	150	617,88 ± 0,09a	880,69 ± 21,94tuv	11,33 ± 3,05k-p
WWF	16	170	617,49 ± 0,26a	777,02 ± 24,44w-aa	5,67 ± 2,08op
CS	12	130	138,62 ± 7,95v	772,91 ± 14,30yzaa	40,67 ± 4,16def
CS	12	150	143,04 ± 9,17uv	535,61 ± 9,89ac	31,67 ± 2,31d-ı
CS	12	170	154,56 ± 6,02uv	824,92 ± 17,83v-z	46,00 ± 8,84d
CS	14	130	269,41 ± 5,19rs	3430,05 ± 26,94b	116,33 ± 9,41a
CS	14	150	205,80 ± 10,95t	1048,20 ± 26,31opq	36,00 ± 5,57d-g
CS	14	170	159,54 ± 9,54uv	724,59 ± 11,86aaab	36,33 ± 6,35d-g
CS	16	130	214,50 ± 8,88t	1330,91 ± 6,96gh	40,33 ± 8,38def
CS	16	150	163,56 ± 2,86u	845,14 ± 16,8vw	48,33 ± 2,52d
CS	16	170	204,17 ± 5,73t	2262,87 ± 17,69e	67,33 ± 7,23c
WWF-CS	12	130	258,66 ± 7,89s	1033,43 ± 18,91pq	39,33 ± 9,50d-g
WWF-CS	12	150	268,08 ± 9,27rs	1096,74 ± 27,19nop	32,00 ± 6,25d-h
WWF-CS	12	170	265,49 ± 8,80rs	1170,75 ± 14,91j-m	31,33 ± 1,53d-j
WWF-CS	14	130	297,70 ± 9,66pq	757,99 ± 27,27zaa	22,00 ± 6,93 g-o
WWF-CS	14	150	300,06 ± 4,87pq	1045,09 ± 8,59opq	22,67 ± 1,52 g-o
WWF-CS	14	170	315,92 ± 7,04op	1125,62 ± 21,51lmn	44,00 ± 2,65de
WWF-CS	16	130	543,68 ± 4,93cde	1034,06 ± 21,55pq	14,00 ± 2,64j-p
WWF-CS	16	150	539,52 ± 8,59c-f	944,74 ± 21,72rst	14,00 ± 5,00j-p
WWF-CS	16	170	544,23 ± 5,13cde	1039,37 ± 11,82opq	16,33 ± 1,50 h-p
WWF-D	12	130	346,53 ± 8,92lmn	1084,76 ± 13,53nop	24,67 ± 1,54f-m
WWF-D	12	150	426,30 ± 8,80j	850,65 ± 20,82uv	13,33 ± 3,52k-p
WWF-D	12	170	548,10 ± 10,16 cd	1183,60 ± 24,74jkl	16,33 ± 3,21 h-p
WWF-D	14	130	617,64 ± 0,47a	854,26 ± 28,07uv	8,33 ± 1,55 L-p
WWF-D	14	150	617,65 ± 0,44a	660,43 ± 28,82ab	7,33 ± 1,49 m-p
WWF-D	14	170	617,40 ± 0,22a	842,30 ± 23,30vwx	7,33 ± 0,58 m-p
WWF-D	16	130	617,68 ± 0,46a	882,32 ± 14,19tuv	6,00 ± 2,00op
WWF-D	16	150	617,21 ± 0,66a	837,41 ± 23,09v-y	7,00 ± 3,47nop
WWF-D	16	170	617,80 ± 0,37a	752,64 ± 16,74aa	4,00 ± 1,01p
CS-D	12	130	147,69 ± 4,03uv	740,53 ± 13,75aa	41,00 ± 1,99def
CS-D	12	150	165,37 ± 7,11u	3285,87 ± 10,76c	125,33 ± 6,43a
CS-D	12	170	198,64 ± 7,28t	1329,88 ± 13,97gh	46,33 ± 8,38d
CS-D	14	130	325,93 ± 6,69no	1367,45 ± 13,54 g	28,00 ± 7,01e-k
CS-D	14	150	296,64 ± 4,40pq	3741,23 ± 19,00a	87,33 ± 6,99b
CS-D	14	170	286,09 ± 8,52qr	1563,18 ± 22,03f	31,00 ± 5,07d-j
CS-D	16	130	514,24 ± 7,062ghı	2567,64 ± 26,89d	39,33 ± 4,53d-g
CS-D	16	150	389,54 ± 7,57k	1609,29 ± 7,05f	25,67 ± 7,51f-l
CS-D	16	170	354,25 ± 9,46 L	1043,33 ± 18,02opq	17,33 ± 2,07 h-p
WWF-CS-D	12	130	358,97 ± 5,91 L	771,30 ± 22,88yzaa	24,33 ± 5,87f-n
WWF-CS-D	12	150	353,48 ± 7,671 lm	955,99 ± 24,96rs	22,00 ± 5,29 g-o
WWF-CS-D	12	170	329,65 ± 9,011mno	954,17 ± 27,86rs	25,00 ± 2,08f-l
WWF-CS-D	14	130	522,65 ± 8,571efg	917,98 ± 20,01stu	13,33 ± 1,16k-p
WWF-CS-D	14	150	495,63 ± 6,161hı	1139,42 ± 10,20k-n	16,00 ± 3,61 h-p
WWF-CS-D	14	170	490,52 ± 9,921ı	1008,85 ± 14,39qr	17,67 ± 3,79 h-p
WWF-CS-D	16	130	614,23 ± 6,13a	1120,53 ± 24,81lmn	9,33 ± 0,58 L-p
WWF-CS-D	16	150	515,82 ± 7,781fgh	1035,74 ± 18,68pq	11,00 ± 1,73k-p
WWF-CS-D	16	170	531,05 ± 7,33d-g	1204,77 ± 13,36jk	10,00 ± 3,46 L-p

*WWF: whole wheat flour extruded product, CS: corn semolina extruded product, D (%5 w/w): D-glucose monohydrate, WWF-CS: (70:30): 70% whole wheat flour and 30% corn semolina mixture

** Different letters show the means in the same column are significantly (*p* < 0.05)

The maximum force value obtained on the texture analyzer determines the resistance of the products to penetration and is defined as hardness [8]. The WWF extruded product, produced at 16% raw material humidity and 150 °C output die temperature, was found to have the highest hardness, while the CS extruded product, produced at 12% raw material humidity and 130 °C output die temperature, had the lowest hardness value. The maximum hardness was found in the WWF-D (558.85) extruded product group and the lowest in the CS (193.80 ± 52.31) extruded product group among the extruded products made with various formulas (*p* < 0.05). D-glucose monohydrate was found to improve hardness across all extruded product groups (Table 4). The hardness of the products was shown to increase with increasing raw material humidity (*p* < 0.05); the effect of the change in output die temperatures was found to be statistically insignificant. Because the bran in WWF-extruded products fills in the gaps in the cell structure and provides a tight structure, it is believed that these products are significantly harder than CS-extruded products. The expansion index and porosity were found to be inversely proportional to hardness. It is thought that the addition of D-glucose monohydrate may be responsible for the formation of products with a tight structure by affecting pore formation. There has been a noticeable increase in hardness as the moisture content of the raw material increases. Stojceska et al. [36] found that the hardness of the extruded products they produced from corn starch was lower than that produced from wheat flour.

### Sensory Analysis Results

**Fig. 1:**
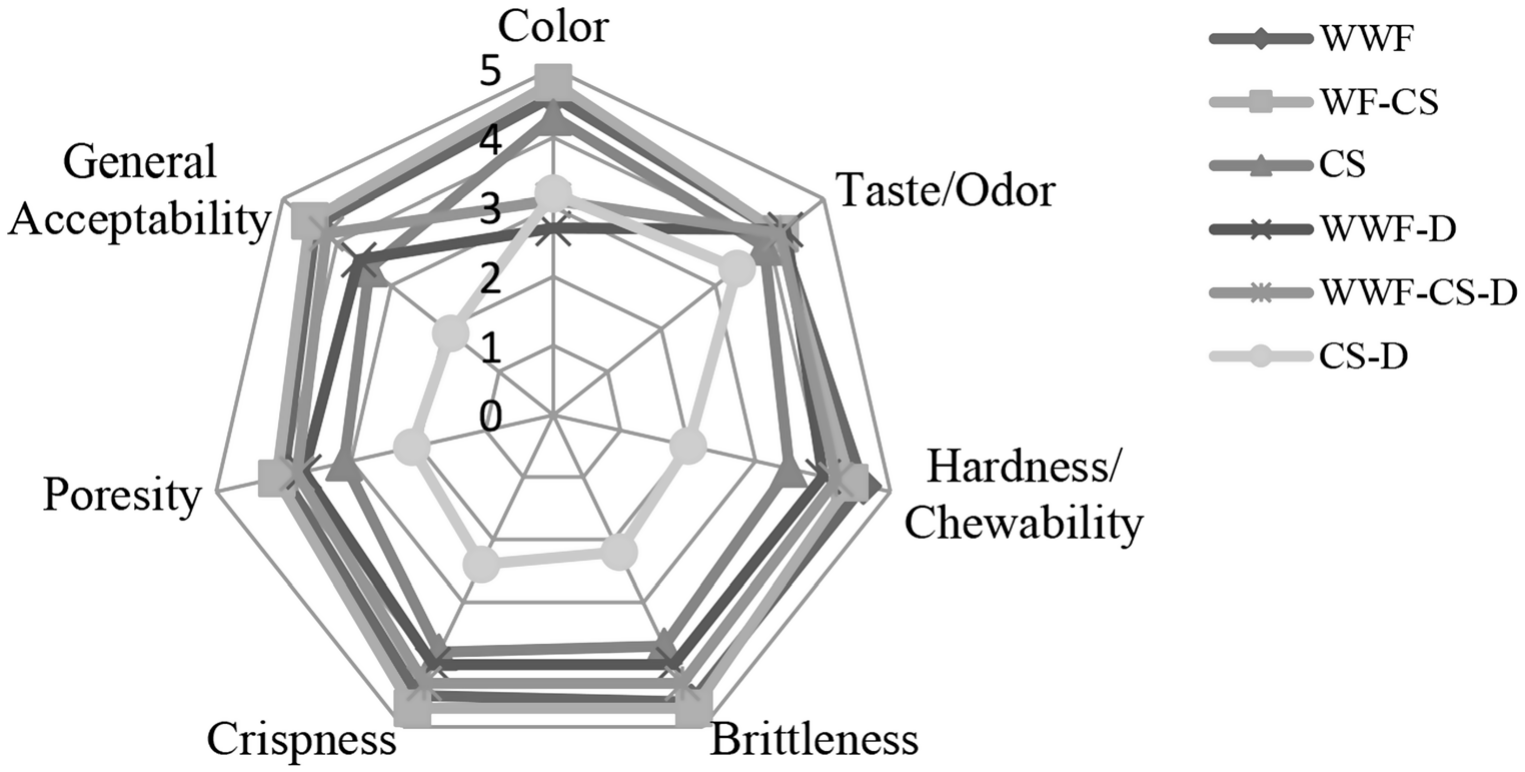
Graph of the sensory analysis results of extruded products produced under 150 °C output die temperature and 12% raw materials humidity conditions

Figure 1 shows that among extruded products in different formulations produced WWF and WWF-CS have the highest values of color, taste/odor, hardness/chewability, brittleness, crispness, poresity, and general acceptability sensory scores. CS-D extruded products were determined to receive the lowest sensory scores. According to the sensory analysis results for extruded products, it was determined that color, taste/odor, hardness/chewability, fragility, crispness, pore structure, and general acceptability parameters were affected by raw material humidity and output die temperatures. WWF-CS extruded products produced at 12% raw material humidity and 150 °C output die temperature were determined to receive the highest sensory scores for general acceptability.

## Conclusion

WWF extruded products are assumed to have a lower expansion index than CS extruded products, with fiber content preventing the product from expanding. The addition of maize semolina was found to improve the expansion index of extruded products, but the addition of D-glucose monohydrate decreased the expansion index. Bulk density and actual density values, which were found to be inversely proportional to the expansion index, were low in CS extruded products, and in extruded products with D-glucose monohydrate addition, they were detected at high values. The bulk density and actual density were found to be lowest at 12% raw material humidity, and density rose as raw material humidity increased. Extruded products with WWF-CS and CS had high porosity, while those with D-glucose monohydrate were determined to have low porosity. It has been determined that the water binding capacities of extruded products increase with the increase in raw feed raw materials humidity and extruder output die temperature, while the addition of D-glucose monohydrate decreases them. WWF extruded products have been found to be firmer, less brittle, and less fragile than CS extruded products, and the textural attributes of WWF-CS products, which are mixtures, are similar to those of CS extruded products. According to the sensory analysis results, the most liked extruded product groups were determined as WWF-CS and WWF, respectively.

In the manufacturing of extruded products, it was determined that three major extrusion working parameters, such as output die temperature, raw material humidity, and raw material components, have an important role. A functional snack product that can be consumed was produced from a mixture of whole wheat flour and corn semolina. It has been determined that the extruded product produced from whole wheat flour can be consumed as a healthy snack in terms of nutrition compared to other oily and additive chip products. This research is expected to serve as an example for future research into the creation of functional products using extrusion technology.

## Data Availability

No datasets were generated or analysed during the current study.

## Abbreviations

WW: Whole wheat flour
CS: Corn semolina
D: D-glucose monohydrate
WWF-CS: Whole wheat flour- corn semolina
WWF-D: Whole wheat flour- D-glucose monohydrate
WWF-CS-D: Whole wheat flour- corn semolina- D-glucose monohydrate
CS-D: Corn semolina- D-glucose monohydrate

## References

[1] Moscicki L, Van Zuilichem DJ (2011) Extrusion-cooking and related technique. Extrusion-Cooking techniques: applications, theory and sustainability. Wiley, Weinheim, pp 1–24. 10.1002/9783527634088

[2] Yağcı S, Doğan F (2016) Ekstrüzyon Yöntemi Ile mercimek (Lens culinaris) Bazlı Glutensiz Bulgur-Benzeri Ürün geliştirilmesi ve Fiziksel-Fonksiyonel Özelliklerinin incelenmesi. Selçuk Tarım Bilimleri Dergisi 3(1):59–67

[3] Samray MN (2018) Enzime dirençli Nişasta İlavesinin Galeta unundan üretilen ekstrüzyon ürünlerinin Fiziksel ve Kimyasal Özellikleri Üzerine Etkisi. Yüksek Lisans Tezi, Hacettepe Üniversitesi Fen Bilimleri Enstitüsü, Ankara

[4] Doğan F (2014) Nohut Bazlı ekstrüde ürünlerin geliştirilmesi. Yüksek Lisans Tezi, Mersin Üniversitesi Fen Bilimleri Enstitüsü, Mersin

[5] Okuroğlu B (2019) Bulgur Altı Ununun ekstrüde Çerez Gıda üretiminde Kullanımı. Yüksek Lisans Tezi. Karamanoğlu Mehmetbey Üniversitesi Fen Bilimleri Enstitüsü, Karaman

[6] Özer EA (2007) Ekstrüzyon Yöntemi İle Besleyici değeri Yüksek Çerez Tipi Fonksiyonel Bir Ürün Geliştirme. Doktora Tezi, Çukurova Üniversitesi Fen Bilimleri Enstitüsü, Adana

[7] Maskan M, Altan A (Eds.) (2012). İntroduction to Extrusion Technology. Advances in food extrusion technology, (p. 130), 18–38. Boca Raton, FL, USA: CRC press.

[8] Çalışkan A (2019) Beta Glukan Katkılı Fonksiyonel Kahvaltılık Gevrek üretiminin Yanıt Yüzey Metodolijisi Ile optimizasyonu. Yüksek Lisans Tezi. Karamanoğlu Mehmetbey Üniversitesi Fen Bilimleri Enstitüsü, Karaman

[9] Zheng X, ve Wang SS (1994) Shear induced starch conversion during extrusion. J Food Sci 59:1137–1143

[10] Moraru CI, Kokini JL (2003) Expansion during extrusion and microwave heating of cereals foods. Reviews Food Sci Food Saf 2:147–165. 10.1016/j.carbpol.2005.09.007 10.1111/j.1541-4337.2003.tb00020.x 33451228

[11] Ajita T (2018) Extrusion Cooking Technology: An Advance Skill for Manufacturing of Extrudate Food Products. Extrusion of Metals, Polymers, and Food Products, (Ed.) Qamar, Z.S. Oman, 198–210. 10.5772/intechopen.73496

[12] Alam MS, Kaur J, Khaira H, Gupta K (2016) Extrusion and extruded products: changes in quality attributes as affected by extrusion process parameters: A review. Crit Rev Food Sci Nutr 56(3):445–473. 10.1080/10408398.2013.779568 25574813 10.1080/10408398.2013.779568

[13] Ding QB, Ainsworth P, Plunkett A, Tucker G, Marson H (2006) The effect of extrusion conditions on the functional and physical properties of Wheat-Based expanded snacks. J Food Eng 73(2):142–148. 10.1016/j.jfoodeng.2005.01.013

[14] Elgün A, Türker S, Bilgiçli N (2015) Tahıl ve Ürünlerinde Analitik Kalite Kontrolü, 32–34, Konya

[15] Nizamlıoğlu NM (2015) Kavurma ve Depolama Koşullarının Bademin Bazı Fiziksel, Kimyasal ve Duyusal Özellikleri Üzerine Etkisi. Doktora Tezi, Pamukkale Üniversitesi Fen Bilimleri Enstitüsü, Denizli

[16] Yavuz M (2014) Effect of added ingredıents on the Acrylamıde level and qualıty of extrudates. Yüksek Lisans Tezi, Orta Doğu Teknik Üniversitesi Fen Bilimleri Enstitüsü, Ankara

[17] Masatcioglu MT, Gökmen V, Ng PK, Köksel H (2014) Effects of formulation, extrusion cooking conditions, and CO2 injection on the formation of acrylamide in corn extrudates. J Sci Food Agric 94(12):2562–2568. 10.1002/jsfa.6598 24497201 10.1002/jsfa.6598

[18] Onoğur TA, Elmacı Y (2011) Gıdalarda Duyusal Değerlendirme. Sidas Medya, İzmir

[19] Tanju Ş, Sümbül Y (1986) Gıda Sanayisinde ekstrüzyonla İşleme Teknolojisi. Gıda Dergisi 11(2):89–94

[20] Miranda RJ, Ruiz LII, Herman-Lara E, Martínez SCE, Delgado LE, Vivar VMA (2011) Development of extruded snacks using Taro (Colocasia esculenta) and nixtamalized maize (Zea mays) flour blends. LWTFood Sci Technol 44(3):673–680. 10.1016/j.lwt.2010.06.036

[21] Wani SA, Kumar P (2016) Development and parameter optimization of health promising extrudate based on Fenugreek oat and pea. Food Bioscience 14:34–40. 10.1016/j.fbio.2016.02.002

[22] Sobota A, Rzedzicki Z (2009) Effect of the extrusion process of corn semolina and pea hulls blends on chemical composition and selected physical properties of the extrudate. Int Agrophys 23(1):67–79. 10.1016/j.foodchem.2008.09.043

[23] Hashimoto JM, Grossmann MVE (2003) Effects of extrusion conditions on quality of cassava Bran/Cassava starch extrudates. Int J Food Sci Technol 38(5):511–517. 10.1046/j.1365-2621.2003.00700.x

[24] Chang YH, Ng PK (2011) Effects of extrusion process variables on quality properties of Wheat-Ginseng extrudates. Int J Food Prop 14(4):914–925. 10.1080/10942910903491173

[25] Sue SL, Sulaiman R, Sanny M, Hanani ZA (2015) Effect of extrusion barrel temperatures on residence time and physical properties of various flour extrudates. Int Food Res J, 22(3)

[26] Singh B, Hussain SZ, Sharma S (2015) Response surface analysis and process optimization of twin screw extrusion cooking of Potato-Based snacks. J Food Process Preserv 39(3):270–281. 10.1111/jfpp.12230

[27] Fan J, Mitchell JR, Blanshard JM (1996) The effect of sugars on the extrusion of maize grits: I. the role of the glass transition in determining product density and shape. Int J Food Sci Technol 31(1):55–65. 10.1111/j.1365-2621.1996.22-317.x

[28] Yuliani S, Torley PJ, D’Arcy B, Nicholson T, Bhandari B (2006) Effect of extrusion parameters on flavour retention, functional and physical properties of mixtures of starch and D-Limonene encapsulated in milk protein. Int J Food Sci Technol 41:83–94. 10.1111/j.1365-2621.2006.01409.x

[29] Korkerd S, Wanlapa S, Puttanlek C, Uttapap D, Rungsardthong V (2016) Expansion and functional properties of extruded snacks enriched with nutrition sources from food processing By-Products. J Food Sci Technol 53(1):561–570. 10.1007/s13197-015-2039-1 26787975 10.1007/s13197-015-2039-1 PMC4711464

[30] Sumargo F, Gulati P, Weier SA, Clarke J, Rose DJ (2016) Effects of processing moisture on the physical properties and in vitro digestibility of starch and protein in extruded brown rice and Pinto bean composite flours. Food Chem 211:726–733. 10.1016/j.foodchem.2016.05.097 27283689 10.1016/j.foodchem.2016.05.097

[31] Thymi S, Krokida MK, Pappa A, Maroulis ZB (2005) Structural properties of extruded corn starch. J Food Eng 68(4):519–526. 10.1016/j.jfoodeng.2004.07.002

[32] Pitts KF, Favaro J, Austin P, Day L (2014) Co-Effect of salt and sugar on extrusion processing, rheology, structure and fracture mechanical properties of Wheat–Corn blend. J Food Eng 127:58–66. 10.1016/j.jfoodeng.2013.11.026

[33] Yağcı S, Göğüş F (2008) Response surface methodology for evaluation of physical and functional properties of extruded snack foods developed from Food-by-Products. J Food Eng 86(1):122–132

[34] Mendonca S, Grossmann MVE, Verhé R (2000) Corn Bran as A fibre source in expanded snacks. LWT-Food Sci Technol 33(1):2–8. 10.1006/fstl.1999.0601

[35] Min W, Yi L, Lijun W, Dong L, Zhihuai M (2015) Effects of extrusion parameters on physicochemical properties of flaxseed snack and process optimization. Int J Agric Biol Eng 8(5):121–131. 10.3965/j.ijabe.20150805.2093

[36] Stojceska V, Ainsworth P, Plunkett A, İbanoğlu Ş (2009) The effect of extrusion cooking using different water feed rates on the quality of Readyto-Eat snacks made from food by-Products. Food Chem 114(1):226–232. 10.1016/j.foodchem.2008.09.043

